# Changing practice as a quality indicator for primary care: analysis of data on voluntary disenrollment from the English GP Patient Survey

**DOI:** 10.1186/1471-2296-14-89

**Published:** 2013-06-25

**Authors:** Shobhana Nagraj, Gary Abel, Charlotte Paddison, Rupert Payne, Marc Elliott, John Campbell, Martin Roland

**Affiliations:** 1 Cambridge Centre for Health Services Research, Primary Care Unit, Institute of Public Health, University of Cambridge, Forvie Site, Robinson Way, Cambridge CB2 0SR, UK; 2 RAND Corporation, 1776 Main Street, Santa Monica, California 90401-3208, USA; 3 Primary Care Research Group, Peninsula Medical School, St Lukes, Smeall Building, St Lukes Campus, Magdalen Road, Exeter EX1 2LU, England

## Abstract

**Background:**

Changing family practice (voluntary disenrollment) without changing address may indicate dissatisfaction with care. We investigate the potential to use voluntary disenrollment as a quality indicator for primary care.

**Methods:**

Data from the English national GP Patient Survey (2,169,718 respondents), the number of voluntary disenrollments without change of address, data relating to practice characteristics (ethnicity, deprivation, gender of patients, practice size and practice density) and doctor characteristics were obtained for all family practices in England (n = 8450). Poisson regression analyses examined associations between rates of voluntary disenrollment, patient experience, and practice and doctor characteristics.

**Results:**

Mean and median rates of annual voluntary disenrollment were 11.2 and 7.3 per 1000 patients respectively. Strongest associations with high rates of disenrollment were low practice scores for doctor-patient communication and confidence and trust in the doctor (rate ratios 4.63 and 4.85). In a fully adjusted model, overall satisfaction encompassed other measures of patient experience (rate ratio 3.46). Patients were more likely to move from small practices (single-handed doctors had 2.75 times the disenrollment rate of practices with 6–9 doctors) and where there were other local practices. After allowing for these, substantial unexplained variation remained in practice rates of voluntary disenrollment.

**Conclusion:**

Family practices with low levels of patient satisfaction, especially for doctor patient communication, are more likely to experience high rates of disenrollment. However substantial variation in disenrollment rates remains among practices with similar levels of patient satisfaction, limiting the utility of voluntary disenrollment as a performance indicator for primary care in England.

## Background

In some countries such as the US, disenrollment rates from health plans are published routinely and are publically available as indicators of the quality of medical care. Although voluntary disenrollment rates have been used as a marker of patient satisfaction, the relationship between voluntary disenrollment and patient satisfaction is complex and multi-factorial [1]. Amongst the factors which influence people to change their primary care provider are the doctor’s interpersonal skills [2], access to care [3] and patients’ ability to choose their primary care provider [4]. In the UK, there is universal access to primary care and near universal registration with a family practice. Patients usually register with a family practice near their home and most often change their family practice because they are moving house. Unlike in the US, there are no financial reasons for changing provider (e.g. changing to a plan with a different type of coverage). People who change practice without changing address in the UK may therefore be expressing dissatisfaction with the care they have received.

In this study, we use the term voluntary disenrollment without change of address to signify patients who voluntarily leave their primary care practice. The percentage of patients leaving their family practice without change of address ranges from 0.4% [5] to 2.1% of the registered population per year [3]. Low rates may reflect high overall satisfaction with family practitioners [6], the limited choice available to patients, or relate to how patients make use of the choices offered to them [7-10]. With the aim of increasing choice and convenience, the government in England has recently established demonstration sites in which patients will be offered much greater choice of practice – e.g. commuters being able to register near work rather than near home [11]. If successful, these pilots will be rolled out more widely. It is not yet clear if this will lead to more patient movements between practices and what will drive these movements.

The aims of this study were to determine whether rates of voluntary disenrollment in England were associated with i) poor patient experience, ii) practice and doctor characteristics or iii) the availability of other family practices in the locality. We also considered whether rates of voluntary disenrollment should be used as a quality indicator to inform health policy.

## Methods

### Data relating to patients changing family practice

The numbers of patients changing practice without change of address between 1st March 2009 and 28th February 2010 (inclusive) were provided by the UK Department of Health. 8,450 family practices in England had at least one patient who changed practice without changing address. Registered practice list size was also provided by the Department of Health for March 2009 and February 2010 for each practice. All analyses were carried out with the practice as the unit of analysis.

### Patient experience and overall satisfaction

Measures of patient experience and overall satisfaction were taken from the 2009/10 General Practice Patient Survey (2,169,718 respondents from 8,362 family practices, response rate 39%) [6]. We then excluded practices with a change in list size of more than 10% during the study year, practices with a mean list size less than 1000, and practices that changed postcode during the study period. This was to exclude closing and merging practices, unconventional practices (such as boarding school practices and specialist clinics) and practices where motivations for disenrollment might have been dictated by special circumstances (e.g. practices moving address or using temporary accommodation). We also excluded 248 practices with fewer than 100 responses on the GP Patient Survey to increase the reliability of patient experience scores. A total of 442,731 voluntary disenrollments without change of address appear in the final data set of 7812 practices.

Case-mix adjusted estimates of practice level scores were calculated for items addressing four components of patient experience: i) communication with doctors and nurses (four questions); ii) access to care (three questions); iii) continuity of care (one question); and iv) overall satisfaction with care (one question). In the case of two communication questions with multiple items, a composite score was calculated as the mean of up to seven sub-items for those patients who answered at least four of the seven. The case-mix adjusted scores were calculated from mixed-effect linear regression models adjusting for patient reported gender, age, ethnicity, deprivation and self-rated health and predict the scores a practice would have received if its case-mix was the same as that of all responders. Full details of the survey and its development have been reported elsewhere [12-14].

### Practice and doctor characteristics

For each practice, registered patient numbers (broken down by sex and age group) were provided by the NHS Information Centre. In addition, data for 2009 for each practice were provided to calculate the number of family practitioners (FP) excluding trainees, the number of patients per full time equivalent FP, the mean number of years since qualification of the FPs in each practice, the proportion of male FPs, and the proportion of FPs trained in the UK for their primary medical qualification. A score for socio-economic deprivation for each practice was calculated by applying the 2007 Lower Super Output Area Index of Multiple Deprivation proportionately to the practice population [15]. GP Patient Survey results were also used to estimate the proportion of Black, Asian, Chinese, mixed race and other non-white patients in each practice.

### Availability of nearby family practices

The geographic location of each practice was determined by matching the practice postcode to data obtained from UK Borders [16]. Using these data, the UK Ordnance Survey national grid reference of the centroid of each of the practice postcodes was obtained. These grid references were used to calculate the number of other practices within 1km of each practice and the number of practices in the same location (co-located practices are interpreted as separate practices operating within a single health centre or building; we assumed this to be the case where the postcode was common to different practices).

### Analysis

Practice-level scores calculated directly from the GP Patient Survey contain measurement error. Using simple scores in regression models may underestimate true effect sizes, and we therefore used shrunken case-mix adjusted estimates of GP Patient Survey scores. This reduced the effects of measurement error and improves the accuracy of estimated effect sizes [17-20]. The annual rate of patients changing practice without change of address was modelled using Poisson regression with a random effect for practice included to account for any remaining variation in disenrollment rates.

In order to compare the strength of association between disenrollment rates and the measures of patient experience, satisfaction, and other patient and practice characteristics, continuous variables (including proportions) were normalised such that the difference between the 5th and 95th percentile of practices was equal to one [1] for patient experience measures, and equal to minus one (−1) for all others. This did not affect the continuous nature of the data, but allowed for direct comparison of the magnitude of effect across different variables. The resulting rate ratios should be interpreted as the relative increase in disenrollment rate associated with moving from the 95th to 5th percentile for patient experience measures, and moving from the 5th to the 95th percentile for other continuous variables. Rate ratios of more than one indicate an association with higher disenrollment rates. Where ordered categorical variables have been used (number of doctors in a practice, number of practices within 1km) the reference category was chosen such that it contained either the 5th or 95th percentile. This allowed the largest rate ratios seen for the categorical variables to be compared directly to the rate ratios for the continuous variables and allowed low scoring practices to be compared to high scoring practices (5^th^ vs 95^th^ percentiles).

Models were run in three stages. First, a series of models including a single fixed effect and the random practice effect were run using single measures of patient experience, patient satisfaction, and practice or doctor characteristics. These models (model set 1) were used to assess the crude association between voluntary disenrollment and each of the factors separately. This shows, for example, how much higher the disenrollment rate typically was in a practice on the 5^th^ centile of overall satisfaction compared to a practice on the 95^th^ centile.

A second model included fixed effects for all measures of patient experience, patient satisfaction, practice case-mix, and doctor characteristics, with a random effect to control for practice (model 2). Model 2 was used to assess the same associations as model set 1, but when adjusting for all other variables; for example, to show how much the disenrollment rate typically increased beyond the variations associated with other factors when comparing practices on the 5^th^ and 95^th^ centiles of overall satisfaction.

A final regression model (model 3) was constructed to assess the suitability of using voluntary disenrollment rate as a quality indicator. This model augmented model 2 with log(capitation), log^2^(capitation) and co-located surgery included in order to explain as much variation as possible. Model 3 was used to assess the variation in disenrollment rates that was not explained by measured characteristics (the random effect).

All analyses were performed in using Stata v11.2 (StataCorp, College Station, Texas, USA) and SAS v9.2 (SAS Institute, Cary, NC, USA).

## Results

### Descriptive Statistics

The mean and median rates of annual voluntary disenrollments were 11.2 and 7.3 per 1000 patients respectively (standard deviation 12.0; inter-quartile range 3.7 to 14.4). The mean and median number of voluntary disenrollments was 56 and 42 patients per practice per year respectively (standard deviation 58; inter-quartile range 24 to 72).

### Regression analyses

The results of the unadjusted regression analyses using a single fixed effect (model set 1) and the model adjusted for practice and patient factors (model 2) are shown in Table 1.

**Table 1:** Predictors of disenrollment - unadjusted (Model 1) and adjusted regression (Model 2)

	**Unadjusted (single fixed**		Adjusted model*	
	**effect) model (Model 1)**		**(Model 2)**	
**GP Patient Survey Items**				
	*Rate Ratio	p-value	*Rate Ratio	p-value
	(95% CI)		(95% CI)	
	5^th^ vs. 95^th^		5^th^ vs. 95^th^	
	percentile		percentile	
Helpful receptionists	1.34 (1.25, 1.44)	<0.001	0.82 (0.75, 0.89)	<0.001
Getting appointment within 2 days	1.62 (1.51, 1.73)	<0.001	1.09 (1.02, 1.16)	0.016
Booking appointment in advance	1.07 (0.99, 1.14)	0.076	1.07 (1.00, 1.14)	0.055
Seeing preferred doctor	0.77 (0.72, 0.83)	<0.001	0.90 (0.84, 0.96)	0.001
Satisfaction with opening hours	1.64 (1.53, 1.76)	<0.001	0.65 (0.60, 0.70)	<0.001
Doctor/patient communication	4.63 (4.35, 4.94)	<0.001	1.03 (0.87, 1.21)	0.782
Confidence and trust in doctor	4.85 (4.56, 5.16)	<0.001	1.26 (1.06, 1.49)	0.011
Nurse/patient communication	1.71 (1.60, 1.84)	<0.001	0.90 (0.84, 0.96)	<0.001
Overall satisfaction	3.91 (3.66, 4.18)	<0.001	3.46 (2.86, 4.18)	<0.001
**Doctors’ Characteristics**				
	Rate Ratio (95% CI) 95^th^ vs.5^th^	p-value	Rate Ratio (95% CI) 95^th^ vs. 5^th^	p-value
	
			percentile	
	percentile			
Mean years since FP qualification	3.58 (3.35, 3.83)	<0.001	1.08 (1.01, 1.15)	0.015
Proportion of male FPs	2.21 (2.07, 2.36)	<0.001	1.00 (0.95, 1.05)	0.807
Proportion of overseas qualified FPs	3.52 (3.35, 3.71)	<0.001	0.96 (0.94, 0.98)	<0.001
Patients per full time equivalent FP	1.88 (1.78, 1.98)	<0.001	0.84 (0.81, 0.88)	<0.001
**Practice Characteristics**				
	Rate Ratio (95% CI)	p-value	Rate Ratio (95% CI)	p-value
	
Number of FPs:				
1^†^	4.29 (4.05, 4.56)	<0.001	2.75 (2.56, 2.95)	<0.001
2	2.92 (2.76, 3.09)		2.10 (1.99, 2.22)	
3	2.03 (1.91, 2.16)		1.68 (1.59, 1.76)	
4	1.52 (1.43, 1.61)		1.42 (1.35, 1.49)	
5	1.23 (1.16, 1.31)		1.19 (1.13, 1.25)	
6-9^‡^	Ref		Ref	
10 or More	0.79 (0.73, 0.86)		0.81 (0.76, 0.87)	
Number of practices within 1km:				
0^†^	Ref	<0.001	Ref	<0.001
1	1.53 (1.44, 1.62)		1.21 (1.16, 1.26)	
2	1.68 (1.57, 1.79)		1.21 (1.16, 1.27)	
3	1.94 (1.81, 2.09)		1.23 (1.17, 1.30)	
4	2.39 (2.20, 2.59)		1.32 (1.24, 1.40)	
5	2.29 (2.09, 2.50)		1.24 (1.16, 1.32)	
6-9^‡^	2.68 (2.49, 2.88)		1.36 (1.28, 1.44)	
10 or More	2.97 (2.61, 3.38)		1.40 (1.27, 1.55)	

* Also adjusted for practice level patient demographics

^†^ Contains 5^th^ percentile

^‡^ Contains 95^th^ percentile

* Rate ratios are the relative increase in disenrollment rate associated with moving from the 95th to 5th percentile for patient experience measures, and moving from the 5th to the 95th percentile for other continuous variables. Rate ratio > 1 implies:

• For patient experience - disenrollment more likely at worse performing practices compared to better practices

• For doctor characteristics - disenrollment more likely at practices with higher values compared to practices with lower values

• For practice characteristics - disenrollment more likely compared to reference category

### Patient experience items

Lower patient experience and satisfaction scores were associated with higher rates of disenrollment in the unadjusted model. In particular, doctor-patient communication and confidence and trust in the doctor (rate ratios up to 4.85) show the strongest associations with disenrollment. When adjusted for the other patient experience items as well as patient and practice factors, most of these associations become weak, the main exception being overall satisfaction (rate ratio = 3.46), which then appears to encompass other markers of patient experience.

### Doctor characteristics

The unadjusted analysis (model set 1) shows that practices with older FPs (increased years since qualification), more male FPs, and more overseas qualified FPs, all had higher rates of disenrollment (rate ratios 3.58, 2.21 and 3.52 respectively). After adjusting for patient survey and other factors (model 2), these rate ratios decrease to levels of little practical significance, suggesting that the high disenrollment rate from these practices can be explained by low scores on patient experience and satisfaction.

### Practice characteristics

**Figure 1:**
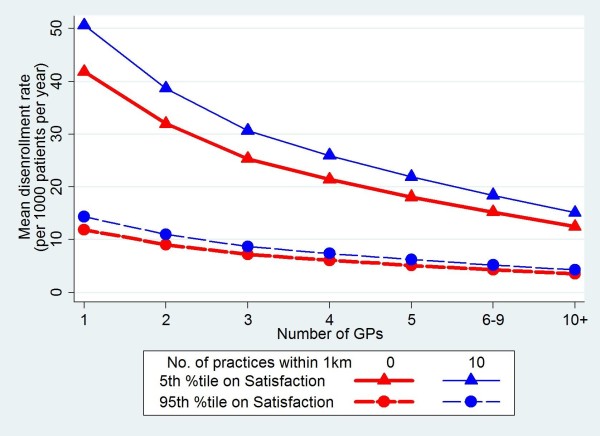
Variation in mean disenrollment rates for different numbers of FPs, numbers of nearby practices and patient satisfaction scores.

Patients were more likely to disenroll from practices with small numbers of doctors and practices where there were other primary care practices nearby (rate ratio of 2.75 for a single handed FP compared to 6–9 FPs in the practice, and rate ratios up to 1.36 for 6–9 surgeries within 1km compared to none) in the adjusted model. These are illustrated in Figure 1 which shows the association between practice size, proximity of neighbouring practices and rates of voluntary disenrollment. The apparent effect of better scores for seeing your preferred doctor being associated with higher disenrollment rates (Table 1) is explained by the fact that this is confounded by the size of practice: continuity of care is, on average, better at practices with fewer doctors, which in turn tend to have higher disenrollment rates.

### The suitability of voluntary disenrollment rate as an indicator of quality

Although we find significant associations between disenrollment and survey and practice characteristics, there is far greater variation between practices due to factors that we have not measured which is captured by the random effect of model 3. Comparing the 95^th^ percentile of this random effect with the 5^th^ percentile produces a rate ratio of 6.22, i.e. the rate of patients leaving a practice without changing address after adjusting for all the factors in our model still varies by over 6 times for the central 90% of practices.

**Figure 2:**
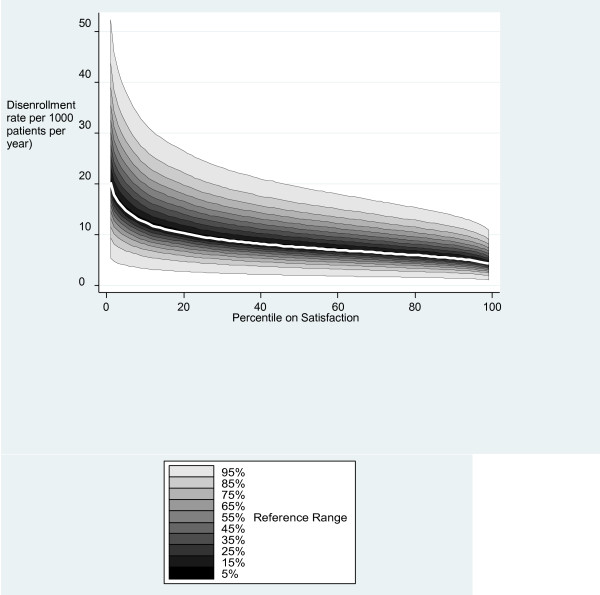
**Variation in adjusted rates of disenrollment for different levels of patient satisfaction.** This figure shows the mean (white line) and the typical ranges of adjusted disenrollment rates (shaded areas) for practices at different levels of patient satisfaction scores. The different levels of shading indicate the fraction of practices which lie within that range. An adjusted disenrollment rate of 10 per 1000 patients per year is within the expected reasonable range for any levels of satisfaction. Disenrollment rates have been adjusted for GP Patient survey items, doctor and practice characteristics.

Figure 2 illustrates the mean rate of voluntary disenrollment and the 95% reference ranges for percentiles of overall patient satisfaction estimated from model 3. Mean voluntary disenrollment rates steadily increase, as patient satisfaction decreases. However, the 95% reference ranges at each percentile are wide and overlap considerably. This makes it difficult to infer patient satisfaction from disenrollment rates and suggests that factors other than satisfaction are of importance in patients’ decision-making regarding disenrollment.

## Discussion

### Voluntary disenrollment, patient experience and patient satisfaction

It has been suggested that voluntary disenrollment could be used as an indicator of patient satisfaction and experience with care [21-24]. The relationship between patient satisfaction and voluntary disenrollment is, however, complex [1]. For example, patients – in particular those with chronic illnesses and the elderly – may prefer to stay with a doctor they know even if their experience of care is not especially good [1,25].

This study highlights aspects of patient experience that were associated with patients changing their family practice. The largest simple associations were with doctor-patient communication, confidence and trust in the doctor, and overall patient satisfaction. A good relationship with the primary care doctor has also been shown in the US to be linked to patient loyalty, and it has been suggested that cultivation of positive interpersonal relationships by the doctor and developing patient trust may be effective strategies to differentiate doctor practices and prevent voluntary disenrollment [26]. Studies in both the US and UK suggest that patients who choose to leave their practice may do so after a breakdown in the doctor -patient relationship [2,3]. The results of our analysis support the doctor-patient relationship as an important factor influencing disenrollment.

In the fully adjusted regression model for all factors which may influence disenrollment rates, patient experience factors were encompassed by a single measure of overall patient satisfaction. If patient experience is viewed as a process, and patient satisfaction an outcome of that process, our results suggest that the various facets of patient experience feed into overall satisfaction with care, the most important of which were doctor-patient communication. This finding is consistent with studies from the USA [21,23,27-29] and also with a previous UK study into the reasons why patients change their primary care practice [10].

Although we have demonstrated an association between voluntary disenrollment and both patient satisfaction and reported experience, there is considerable unexplained variation in rates of voluntary disenrollment between practices. This unexplained variation is much larger than that associated with overall satisfaction (illustrated in Figure 2) indicating that rates of disenrollment cannot easily be interpreted as a quality indicator. For example, a practice with a disenrollment rate of 10 per 1000 patients per year could lie in any of the centile groups for overall satisfaction. For these reasons we do not recommend voluntary disenrollment rates as a quality indicator for primary care practices in the UK.

### Patient choice

After patient experience, we found that practice factors were the next strongest predictors of voluntary disenrollment, and in particular the number of other doctors in the practice (disenrollment was commoner in small practices) and the availability of other nearby practices. Both these factors effectively give patients greater choice, one giving more choice of doctor within a practice, the other more choice outside of the practice. However, the size of the effect of these variables was less than the effect of overall patient satisfaction.

Low overall rates of voluntary disenrollment in England may indicate that satisfaction with care which is generally very high [6], or may be because patients find it difficult to change practice, e.g. practice lists are sometimes ‘closed’ to new patients. Some previous research suggests that people value having choice even though they may not necessarily want to exercise that choice [7,30,31]. However, a recent review of patient choice [32] suggested that a substantial proportion of people (45% of patients studied) would be willing to change to an alternative (e.g. non-local) primary care provider whose characteristics better suited their preferences. A belief that patients want more choice underlies the current UK government’s policy to give patients much greater choice of primary care practice, including a practice which is near their work rather than their home.

### Strengths and limitations of this study

The strength of this study lies in the use of a large data set, and the use of a validated, widely distributed patient questionnaire. To our knowledge, data comparing patient satisfaction with voluntary disenrollment have not previously been reported in England. Limitations of this study include the lack of patient-level data on disenrollment, which would allow more in-depth study of individual patient movements between practices. Our models also assume that individuals act autonomously when leaving their practice whereas it is likely that patients sometimes leave as a family group or with their partner. Other limitations include the limited nature of our measures of patient choice and the 39% response rate of the GP Patient Survey questionnaire in the year 2009–2010, which although comparable with other large public sector surveys, may not be fully representative of the population. The survey is population based and does not depend on a recently practice visit. However, fewer than 1% of respondents had not visited their practice and many of these gave non-informative responses which were excluded from the analysis. Inclusion of this group of patients is therefore unlikely to have influenced the results in any significant way.

## Conclusion

Family practices with low levels of patient satisfaction, especially for doctor patient communication, are more likely to experience high rates of disenrollment. However substantial variation in disenrollment rates among practices with similar levels of patient satisfaction limits the utility of voluntary disenrollment as a performance indicator for primary care in England.

### Permissions

Ethical permission was not required for these analyses.

## Competing interests

The research described here was funded in part by a grant from Ipsos-MORI who developed and delivered the GP Patient Survey for the Department of Health in England. Professor Roland and Professor Campbell contributed to the development of the survey as academic advisors, and their universities received payment for this advice from 2009–2011.All authors declare that they no competing interests.

## Authors’ contributions

All authors contributed to the conception and design of the study. The analyses were carried out principally by SN, GA and ME. All authors contributed to drafting and revising the paper. All authors read and approved the final manuscript.

## Pre-publication history

The pre-publication history for this paper can be accessed here:

http://www.biomedcentral.com/1471-2296/14/89/prepub
